# Symposia Abstracts

**DOI:** 10.1080/24740527.2018.1476310

**Published:** 2018-05-21

There is little question that health research is undergoing a paradigmatic shift with an increasing emphasis on stakeholder involvement, knowledge translation and knowledge mobilization. Entry into non-traditional research areas is often a necessity for widespread knowledge mobilization and represents unchartered territory, often with expertise developing by trial and error. The goals of this symposium are to: a) shed a new light on the role of partnerships with patients and other stakeholders in patient oriented research (not only for the purposes of knowledge translation and knowledge mobilization) as this role is often misunderstood; b) demonstrate how diversification of research expertise can become a necessity in the quest for implementation of widespread and permanent clinical change. Ways of effectively working with patient/stakeholder partners and of diversifying one’s research expertise (mid-career or later) in order to address obstacles to implementation of evidence-based practices will be presented.

## Speaker 1 Abstract Title: Nothing about us without us: Adding the patient voice to Canadian chronic pain research to optimize health practices and patient outcomes

**Speaker 1:** Mary Brachaniec, BScPT, MAHSR, Patient Partner, Canadian Pain Network, Riverview, NB, Canada, mary.brachaniec@gmail.com, @brachaniec_mary

**Speaker 1 Abstract:** The Canadian Institutes of Health Research (CIHR) Strategy for Patient-Oriented Research (SPOR) focuses on research most relevant to patients and on timely implementation of findings to optimize health practices and outcomes. Patients are engaged as active partners working with other stakeholders in all aspects of the research cycle, including governance and oversight, research priority setting, project planning, and Knowledge Translation (KT) activities. The Chronic Pain SPOR Network (CPN) supports high impact Canadian research aiming to unlock the mysteries of pain and to elucidate effective treatment approaches for people with persistent pain. Each CPN Patient Partner brings his/her unique blend of experiences of living with chronic pain and in seeking treatment to improve quality of life and the ability to participate fully in society. This presentation will outline the role of one of our CPN patient partners, who serves as CPN KT Committee Co-chair, and as the first patient partner for the Pain in Older Adults Research team. This evolving role is a particularly good fit for her as caregiver for family members with dementia and persistent pain. Bringing the patient perspective to our research team helps ensure that the voice of this vulnerable population is heard. While patient engagement in health research is gaining momentum internationally, it is reasonably new to the Canadian pain research community. Exploring the opportunities and challenges faced by this CPN patient partner so far can elucidate next steps in moving much-needed evidence into practice to improve the lives of countless Canadians with chronic pain.

## Speaker 2 Abstract Title: The necessity of mid- to late-career research expertise diversification for achieving widespread clinical change: An example from the study of pain in dementia

**Speaker 2:** Thomas Hadjistavropoulos, Ph.D., FCAHS, Department of Psychology and Centre on Aging and Health, University of Regina, Regina, SK, Canada. Thomas.Hadjistavropoulos@uregina.ca, @URHealthPsycLab

**Speaker 2 Abstract:** A program of research on pain assessment in dementia serves as an illustration of the necessity of and entry into research diversification to achieve implementation goals. The research program begun with a series of largely lab-dependent investigations of pain behaviours characteristic of people with dementia. It progressed with development of clinically useful tools for pain assessment at the front line. Following convincing demonstrations of the validity and clinical utility of these tools, the program resulted in international interdisciplinary guideline development. Implementation of clinical solutions, using implementation science methodologies, succeeded in varying degrees but not on a large scale basis. Implementation challenges were largely related to resource barriers and insufficient front-line staff education on cutting edge assessment. Efforts to affect province-wide policy change were met with resistance largely due to concerns around cost and resources. Problem solving to implement change necessitated entry into areas that can reasonably be described as ‘not-traditional” for a group of clinical scientists. These areas included, but were not limited to public policy research, development of automated computer vision technologies to address human resource limitations, estimation of the cost of pain in long-term care, and development of web-based platforms to educate rural front line staff in cutting edge assessment. The transition into these areas was rewarding and necessitated unlikely partnerships. A case will be made that, given increased emphasis on knowledge transfer and mobilization, mid- and late career research expertise diversification is a necessity for the implementation of successful and widespread evidence-based changes in clinical practices.

## Speaker 3 Abstract Title: Digital health technologies to improve pain in young people: Opportunities and challenges for implementation

**Speaker 3:** Jennifer Stinson, RN-EC, PhD, CPNP, FAAN, Research Institute, SickKids and Lawrence S. Bloomberg faculty of Nursing, University of Toronto, Toronto, Ontario, Canada, email: Jennifer.stinson@sickkids.ca, @DrJenStinson

**Speaker 3 Abstract:** This presentation will highlight the strategies our lab used to gain the expertise in digital health technologies (i.e., how to employ a user-centred design approach to ensure patients are actively engaged in all phases; developing a common language across key stakeholders including mobile developers, researchers, clinicians and patients; and cultivating partnerships to ensure wide scale adoption and sustainability). Pain is common across all types of childhood cancers and negatively impacts health-related quality of life (HRQL). Despite available and effective pain treatments, cancer pain remains undermanaged. Children as young as 8 years are able to use electronic diaries to provide reliable and valid data; yet, no diary has been developed to capture the pain experience of pediatric cancer patients. Our lab has developed two real-time multidimensional cancer pain smartphone apps; Pain Squad that tracks cancer pain and Pain Squad+ that tracks and provides just-in-time pain management advice. This presentation will briefly outline the steps in the development and evaluation of the usability (i.e., easy to use, understandable), feasibility (i.e., high adherence and acceptability, few technical difficulties) and psychometric properties of the Pain Squad app (convergent and discriminant validity, internal consistency and responsiveness) and the feasibility and effectiveness of Pain Squad+. The Pain Squad App is now freely available in the Apple store. This presentation will highlight the barriers and challenges in the development, evaluation, implementation and sustainability of mobile pain apps and discuss lessons learned.

**Learning Objective 1:** To familiarize participants with a vast array of expertise and partnerships that are often needed as part of efforts to implement new evidence-based approaches

**Learning Objective 2:** To illustrate an approach toward meaningful engagement with patients as research partners

**Learning Objective 3:** To illustrate, through specific examples from research with older adults and with children, how the necessity of expertise diversification was met and addressed by successful research groups.

